# The Mutual Relations and Consequent Mutual Duties of the Medical Profession and the Community

**Published:** 1860-06

**Authors:** N. S. Davis

**Affiliations:** Professor of Principles and Practice of Medicine in Lind University, and of Clinical Medicine in Mercy Hospital, Chicago


THE CHICAGO
MEDICAL EXAMINER

Vol. I. JUNE, 1860. No. 6

### ORIGINAL COMMUNICATIONS.

*The Mutual Relations and consequent Mutual Duties of the Medical Profession and the Community: A popular Lecture before the Illinois State Medical Society, and the Citizens of Paris, Edgar County, Ill., during the Annual Meeting of the Society, in May*, 1860. By N. S. Davis, M. D., Professor of Principles and Practice of Medicine in Lind University, and of Clinical Medicine in Mercy Hospital, Chicago.

Citizens and Members of the Profession:—The relations of the medical profession to the community at large, are more universal, more intimate, and more important, than those of any other profession or calling among men.

The universality of those relations is founded on necessities the most imperious and delicate. For though many members of the community affect to despise and deride the profession while they are in the enjoyment of health, yet the number who do not earnestly call for medical aid when overtaken by sickness, is exceedingly small. Indeed, it is highly probable that the whole number who have lived in our country from their birth to their final exit from this scene of life, without receiving more or less direct service from the physician, is not equal to those who have committed suicide, or died from sudden violence.

The relations of the profession, extend, however, much beyond the mere personal attentions to the sick. It is to investigations pursued almost solely by the profession, concerning the *causes* of disease, the circumstances under which they act, and the means for their removal that we owe all the valuable sanitary measures of the present day. The improvements in the construction and ventilation of dwellings; the sewering of cities; the quarantine regulations for the protection of seaport towns; the more ample and humane provisions for the Insane; the direct and positive prevention of loathsome and most fatal epidemics, such as the small-pox; and the preparation of provisions and fruit for long voyages in such a manner as almost to banish the scurvy from the ships of enlightened nations, have all resulted, directly or indirectly, from the developments of medical science, and the influence of its legitimate cultivators. Hence it will require but little reflection, to perceive the influence of the science and practice of medicine upon every class, sex, age, and individual belonging to the community, whether at home or abroad—on the land, or the ever restless ocean.

That the relations sustained in the direct intercourse of the physician with his patient are of the most intimate and confidential nature, needs no illustration.

The family physician is admitted to the innermost circle of human society. He comes not only to the hearth-stones, but to the hearts of his patients. As the intelligent instrument for alleviating their sufferings, he necessarily becomes cognizant of their ills, their deformities, and even their mental and moral obliquities. The confessional does not bring the spiritual adviser into closer or more confidential relations with erring mortals, than do the daily duties of the physician.

From the number and variety of relations existing between the profession and the community, and the close personal character of some of them, their paramount importance can be easily deduced. To say that the characters, happiness, health, and even lives of unnumbered individuals depend directly on the intelligence, integrity, and skill of individual members of the profession, is to assert a fact apparent to all. Indeed so fully is the importance of this direct relation between the physician and his patient recognised, that it often causes the more remote and general relations of the profession to the community, as a whole, to be entirely overlooked.

And yet, the latter, when justly estimated, as much outweighs the former in importance, as the *prevention* of disease is preferable to its removal after it has actually laid hold on its victim. To realize the truth of this, compare modern London, with its sewered streets; its supply of water; its detailed sanitary regulations founded directly on knowledge furnished by the regular profession; and the annual ratio of one death in *forty* of its teeming population, with the London of the first half of the seventeenth century, before it was literally destroyed by the great plague and the great fire; and when the ratio of deaths averaged one in *twenty* annually.

Bear in mind that what is true of London, is equally true of almost every city in christendom. Again, look at the history of those twin scourges of the human race, the Plague and Small-Pox, while they were annually *decimating* the population of cities, towns, nay whole provinces, previous to the dawn of the eighteenth century, and compare it with the almost entire exemption from these diseases enjoyed by the inhabitants of christendom at the present time.

Or, turn to that most unfortunate portion of the human race, the insane. Only a few generations have passed since you might have found many thousands of this class in jails, and poor-houses, bound with chains, and regarded as literally "possessed of the devil." But now how great the change. Instead of chains, and prisons, and demons, scarcely a civilized country exists, that is not dotted over with elegant and hospitable asylums, not only for their safe keeping, but for their actual care.

These illustrations might be greatly extended, were it any part of my object to show what or how much the medical profession has done for the prevention and amelioration of human suffering, but at present I simply wish to remind you of the true nature and importance of the relations sustained by the physician to the community, that we may the more readily comprehend the mutual duties growing out of them. From the foregoing observations, it is clearly seen, that the community are dependant on the votaries of the science and art of medicine, not only for the careful treatment of diseases, and the very general prevention of some of the most dreaded, as Scurvy and Small-Pox; but also, for all that knowledge in relation to the causes of disease, the circumstances under which such causes are engendered, and the means for counteracting their effects or procuring their removal, by which the municipal governments of the present day are enabled to devise and execute improvements and sanitary regulations of the greatest importance to the welfare of whole communities.

With relations so numerous, so intimate, and so important, there necessarily arise mutual duties, and obligations of corresponding magnitude, which it is very desirable to have clearly appreciated.

These duties on the part of the Physician, may be embraced in the two following propositions:

1st—Every individual who presumes to discharge the duties of a physician, is under an imperative obligation to obtain such an education as will enable him to give each of his patients, and the public at large, all the benefits that the present state of medical science will afford.

2d—He is under equal obligations, so long as he continues in the profession, to bestow upon its cultivation and application in the treatment of disease, his earnest and undivided attention.

The abstract truth of the first of these propositions, all will admit; yet very few will comprehend the nature and extent of the education which it requires.

Medical science is not composed of a few simple abstract or fixed rules, capable of application in the treatment of all diseases; neither is disease itself a unit, characterized by fixed and unvarying symptoms, and tending always to the same results. On the contrary, man, the last and noblest of the Creator's works—the climax of animated beings; combines in himself almost every variety of structure and function to be found in the whole kingdom of organized living matter; and he lives and moves in the midst of all the varying influences emanating from the earth on which we tread, the air we breathe, and the intellectual and moral forces with which we come in contact. Every elementary cell and fibre of this delicate and complex organism are possessed of properties and functions capable of being more or less influenced by all the exterior agencies that surround us. When these properties and functions are in their natural condition, and the exterior forces and agencies that act upon them are in their natural degree of intensity, health and physical happiness exists. And as disease is a deviation, either in structure or function, or both, from this normal condition, so its nature, tendencies and results, will vary with every variation in the condition under which it is produced. Hence, to study one single disease in all its relations of causation, nature, tendencies, results, and means of prevention and cure, necessarily involves a study of all those changes in, or emanations from, the earth, the water, and the air, with which we are in daily contact; the intimate structure and functions of each part of the human system with the changes they are capable of undergoing; the special laws that govern the various forms of morbid action; and the nature and effects of all such substances or influences as are capable of acting as remedial agents.

It is thus rendered evident to the intelligent mind, that medical science properly so called, is but an aggregation of materials gathered from every other science known to man. For the causes of disease we must draw largely from the facts of Geology, Meteorology, Climatology, and the laws of vegetable growth and decomposition. For a knowledge of the nature and tendencies of disease, we must absorb all the facts of Anatomy and Physiology, human and comparitive, healthy and morbid. For a knowledge of remedies for the modification and cure of disease, we must take largely from Chemistry, organic and inorganic; Botany, Mineralogy, Natural History, and Mental Philosophy. Hence, medical science is the climax —the grand culmination of all sciences, in such a manner that the well disciplined mind may seize upon their almost countless facts and principles, and render them subservient to the highest and noblest of earthly purposes, namely, the prevention and amelioration of human suffering. With what astonishment has the world looked on while men of science have literally chained the electric currents—the "lightnings of heaven," —and made them swift messengers of communication between the remotest parts of the earth. But is it not equally wonderful and far more beneficent to see the educated physician render the same subtle agent subservient to the restoration of palsied limbs, or the resuscitation from temporary death?

If the hasty glance we have taken of the nature and extent of medical science is correct, it is easy to perceive that instead of being fixed and definite in its limits, it is ever progressive—ever expanding—either aggregating new material or dismissing that which has become useless, with every advance that is made in the various departments of human learning. With such a view of the vast field of medical science, with its capacity for unlimited improvement, in strict coincidence with the advance of all the other departments of human knowledge, how insignificant are the contracted dogmas that constitute the basis of the *isms* and *pathys* of the day?

The adage "heat is life and cold is death," constituted the foundation of Thompsonianism, from which has sprung the more modern Botanics and Eclectics.

"Similia similiabus curantur," or like cures like, is equally the basis on which repose all the followers of Hahneman. The full meaning of both may be as well comprehended in an hour as by the study of a year. They admit of neither contraction nor expansion; but are like the procrustean bed, to the iron bars of which all else must be made to fit. If the facts developed by every day's observation prove too long, they must be cut off; if too short, they must be stretched by the imagination until they seem of the right length.

If the nature and extent of true medical science is such as we have indicated, the second duty we have announced as binding on the physician, needs no comment. That man who educates himself in such a way, that he is capable of wielding the vast stores of knowledge embraced in the science and art of medicine, with the highest degree of skill, and keeps himself master of the annual accretions to that store, will find no time or opportunity to distract his attention with other pursuits. If he bestows every waking moment, except such as are required for the direct worship of his maker, on the cultivation of medical science, and its application to the prevention and cure of disease, when he has arrived at the end of full three score years and ten, he will be conscious of having entered but little way into the vast field which was opened out before him at the commencement of his career. Hence brethren of the profession, you who have taken charge of the sick; who have assumed the responsibility of conservators of the public health; you who have unfurled the broad banner of humanity and enlisted for the warfare with the king of terrors, we entreat you not to prove faithless sentinels, sluggishly slumbering on your posts of duty while the archives of our noble science lie moulding on your shelves; or undisciplined soldiers allured after the passionate goddess of partisan politics, or the more gaudy and still more fickle god of wealth, while the grim-monster seizes without resistance the helpless ones who had confided their all to your protection. Nothing is more certain than that no one man can do justice to two or three diverse pursuits at one and the same time.

And while we would have every physician sufficiently familiar with the affairs of government and the various interests of society, to perform all the duties of a good citizen, we would have him never loose sight of the old and true saying, that "no man can serve two masters," for if he does not actually love the one and despise the other, he will at least in cleaving to the one *neglect* the other.

But if the profession of medicine bears relations to the dearest interests of individuals and society, so numerous and important; if its very nature imposes upon its members the duty of acquiring an education the most comprehensive and profound, and the life long performance of services to the suffering that cease not with the summer's heat or the winter's cold—the sunshine or the storm; the noon-day or the midnight; so too does it impose in return obligations no less imperative and important upon the whole community—obligations, indeed, which we fear very few, even of the more enlightened members of society, either seriously consider or acknowledge.

Foremost among the duties that devolve on the community in its relations to our profession, is that of affording to the student of medicine every facility for acquiring that education to which we have already alluded; and to the practitioner equal facilities for accumulating all those facts which will increase our knowledge of the causes, tendencies, and results of diseases. When we remember how closely the dearest interests of every individual are connected with his access to a skillful physician, the pressing necessity for whose services is liable to occur at any and every hour of human life, we should naturally infer that, next to the common school and the place of religious worship, adequate provision for ensuring the careful and thorough education of medical men, would receive the attention of every civilized community. And yet, instead of this we find in almost every community, positive and serious barriers directly in the way of such acquirements.

For instance, not a bone can be set when displaced, not an artery tied, nor a knowledge of the seat and progress of any internal disease obtained, without a good knowledge of the anatomy of the human body.

And no adequate knowledge of anatomy can be gained without actual dissections. But the community, instead of making any provision for facilitating such dissections, have made it an actual crime to procure a single body for that purpose. No one can read the story of the Egyptians exacting of the poor Israelites the manufacture of the full *tale* of bricks, and at the same time refusing them the straw necessary for such manufacture, without feeling a strong indignation at their injustice; and yet the enlightened people of Illinois are guilty of precisely the same species of absurd injustice towards the medical profession. They have enacted laws by which every physician and surgeon are held responsible for all damages resulting from want of skill in the performance of their professional duties. If a limb is broken or dislocated, and the attending surgeon, through ignorance of anatomy, fails to dress it properly, he is held liable for all the damages resulting from such ignorance. Yet they neither provide the surgeon a single body from which he may acquire the desired anatomical knowledge, nor allow him to provide one himself, without being subject to legal prosecution and disgraceful penalties. The great States of New York and Massachusetts have relieved themselves of this absurd treatment of the profession, and gross violation of the interests of the whole people, by the enactment of liberal and just laws for the study of human anatomy. Will not the people of Illinois require their next legislature to imitate the example of the enlightened states we have just named?

The accomplishment of this requires no one to surrender the lifeless remains of his kindred or friends to the mutilations of the dissecting knife, nor does it necessitate the slightest desecration of the burial place for the dead. There are in this and every other State of our glorious Union, plenty of individuals who, while living are supported on public charity, while sick receive freely the gratuitous services of the profession, and when dead have not a friend to ask for their burial. Can there be a rational objection urged against delivering the bodies of such, under proper regulations, to the profession for the sole purpose of extending and perfecting that knowledge of human anatomy, which is so essential to the welfare of every class of the community?

Nor is this all. But all classes of the community should more freely and cheerfully allow, not dissections, but simple *post mortem* examinations of the bodies of those who die; so that the practicing physician may more frequently compare the actual progress and results of disease with the symptoms during life. By such comparisons, frequently and carefully made, he would be far better able to rightly understand the meaning of the symptoms during sickness; to more clearly anticipate certain tendencies; and correspondingly better able to successfully ward off fatal results.

Enlightened self-interest alone, should prompt every individual to be far more liberal in all these matters. There is a wide spread error in the popular mind, which I will not pass by in this connection. It is this: Whenever members of the medical profession ask the community or the Legislature to allow post mortem examinations, or legalize dissecting, or to enact laws for the protection of members of society against knavish empiricism and reckless ignorance, the idea is at once entertained that these things are asked for to accommodate and benefit the profession and not the community. Nothing could be farther from the truth. For, delving for knowledge into the offensive and decaying structures of a lifeless body is not a pleasant *pastime* for the physician, nor are his pecuniary interests increased in proportion to his acquisition of knowledge. On the contrary, it is quite probable that medical men, even in the dark ages of the world's history, were better rewarded for their services, however worthless they might have been, than they are now in the most enlightened portions of Christendom. No, fellow-citizens ! it is you and not the profession that reap the benefits of every advancement made in any department of medical science. The reward of the profession was just as good, and the demand for its services far greater, before Jenner discovered that vaccination would prevent that loathsome scourge, the small-pox, than it is now. The labor and skill of the operating surgeon must be just as great now, when his patient lies unconsciously smiling under the influence of the pleasant dreams produced by Ether or Chloroform, as formerly when the poor creature writhed in keen anguish beneath every stroke of the knife. Yet where is the mathematician who can estimate the number of lives saved, or the amount of human suffering prevented by these two simple discoveries?

Another important mode of advancing medical knowledge, and therefore another duty devolving on the community, consists in the enactment and strict enforcement of laws for registering all births, marriages and deaths. By such records, when faithfully kept, the facts are afforded for ascertaining the duration of human life, and the ratio of mortality in every town and precint in the State. By comparing these with the Geological, Topographical, and Climatic conditions of each locality, the profession would be enabled to throw much additional light on the causes of disease, and the means for their removal; thereby reflecting still further benefits upon the community at large.

Still another important duty which the community have hitherto neglected, consists in the conferring upon well educated physicians such offices, as from their nature require the possession of sound medical knowledge for the proper discharge of the official duties appended thereto. Such is the important office of *Coroner* in each county. One of the chief duties of Coroner consists in holding inquests upon the bodies of persons who come to a sudden or violent death, for the purpose of ascertaining the cause of such death, and developing all the facts that may bear upon the question of crime or aid in its detection and punishment. From the very nature of the investigation, none but a well educated medical man is competent to the task. I do not mean that this or any other office should be conferred on such members of the profession as will neglect their appropriate duties, and stoop to do the greatest amount of mere political partisan work, but the offices of Coroner and that of physician to the county poor, should be conferred on such members of the medical profession, and such only as are qualified by their education and their high moral integrity to do full justice both to the community and the suffering poor. Finally, fellow citizens you cannot fully discharge, not only your duty to the profession, but to yourselves and humanity at large, without on one hand discouraging every species of charlatanism and quackery, and on the other liberally and cordially sustaining every effort that is made to advance either the science or the art of legitimate medicine. And have not the great mass of the regular profession, throughout all ages of the world shown themselves entitled to the confidence and respect of mankind?

Have they, either individually or collectively, shown themselves illiberal, selfish or unfaithful to the high trust reposed in them? On the contrary, have they not always, through all ages, freely and cheerfully bestowed their services upon the unfortunate poor, not only without pecuniary reward, but often without the poor return of a "thank you Sir"? In all the other departments of human life, the universal maxim is, that "the laborer is worthy of his hire." But I say proudly and fearlessly that even suffering poverty never appealed to our profession for help without a liberal response. And there are those in the profession of our own State who give to the poor freely, time and labor worth from one to two thousand dollars annually.

Not only is our profession a liberal one in its responses to the calls of suffering humanity, but its unselfishness is displayed in the fact, that every improvement or discovery made by any one of its members, must at once become the common property of the whole. In the various arts and callings of life, if a man makes an important discovery or useful invention, his first object is to secure a patent by which he restricts to himself such pecuniary profits as the importance of the invention will develope. But let a member of our profession attempt thus selfishly to restrict, by patent right, the use and profits of any discovery or improvement calculated to ameliorate human suffering, and he is at once scouted from all fellowship with his brethren; it being a law of the profession stronger than any legislative enactment, that the interests of humanity are paramount to the pecuniary considerations of any individual.

In regard to the faithfulness of the profession in times of danger, let the history of their conduct during a thousand epidemics answer. The soldier faces danger and death upon the battle-field, because he is supported on either side by his comrades, with his foes in full view before him, and the loud plaudits of his countrymen already ringing in his ears.

But when the unseen angel of death is hovering over a community in the form of an epidemic of cholera, yellow fever, &c., snatching a child here, a mother there, and a father yonder, staying its fatal work neither at midnight nor at noonday, until the badge of mourning decorates the door handles on every block, and the gloomy hearse is met at every street corner, who is it that stands firm when others flee? Who is it, that, in such times, may be seen in the still hour of night, or under the burning sun of noon-day, wending his way alike into the decorated mansions of the rich, or the narrow alleys and by-ways where the poor congregate, and in both, with steady hand and stout heart, administering medicine to the sick, words of consolation to the afflicted, and expressions of hope to all? Ah! it is the unpretending, the care-worn, but the intelligent and faithful practitioner of the healing art. Brethren of the profession, when I look at the nature of our exalted and beneficent vocation—when I survey the great field we cultivate, in which the most sublime facts of science, and the direst sufferings of humanity are mingled together—and above all, when I look at the calm, silent, heroism of our brethren as displayed on such occasions as have been presented at Sandusky, Norfolk, Portsmouth, and a thousand other localities, I confess that I am proud of the name of *Physician.* And I am ready to exhort you to go on, *faithful* to your chosen calling, regardless of the smiles or frowns of those around you, until at last you reach that field on which the grim-messenger will never enter.

